# New Estimations for Shannon and Zipf–Mandelbrot Entropies

**DOI:** 10.3390/e20080608

**Published:** 2018-08-16

**Authors:** Muhammad Adil Khan, Zaid Mohammad Al-sahwi, Yu-Ming Chu

**Affiliations:** 1 College of Science, Hunan City University, Yiyang 413000, China; 2 Department of Mathematics, University of Peshawar, Peshawar 25000, Pakistan; 3 Department of Mathematics, University of Sa’adah, Sa’adah 1872, Yemen; 4 Department of Mathematics, Huzhou University, Huzhou 313000, China

**Keywords:** Jensen inequality, convex function, Shannon entropy, Zipf–Mandelbrot entropy, 26D15

## Abstract

The main purpose of this paper is to find new estimations for the Shannon and Zipf–Mandelbrot entropies. We apply some refinements of the Jensen inequality to obtain different bounds for these entropies. Initially, we use a precise convex function in the refinement of the Jensen inequality and then tamper the weight and domain of the function to obtain general bounds for the Shannon entropy (**SE**). As particular cases of these general bounds, we derive some bounds for the Shannon entropy (**SE**) which are, in fact, the applications of some other well-known refinements of the Jensen inequality. Finally, we derive different estimations for the Zipf–Mandelbrot entropy (**ZME**) by using the new bounds of the Shannon entropy for the Zipf–Mandelbrot law (**ZML**). We also discuss particular cases and the bounds related to two different parametrics of the Zipf–Mandelbrot entropy. At the end of the paper we give some applications in linguistics.

## 1. Introduction

The idea of the Shannon entropy [1] plays a key role in information theory, while in some cases, it is denoted as measure of uncertainty. There are basically two methods for understanding the Shannon entropy. Under one point of view, the Shannon entropy quantifies the amount of information in regard to the value of X (after measurement). Under another point of view, the Shannon entropy tells us the amount of uncertainty about the variable of X before we learn its value (before measurement) [2]. The random variable, entropy, is characterized regarding its probability distribution and it can appear as a better measure of predictability or uncertainty. **SE** permits the appraisal of the normal least number of bits expected to encode a series of symbols based on the letters in order of estimation and the recurrence of the symbols. The formula for **SE** is given by [1]

(1) $$S\left(\Psi \right)=\sum_{i=1}^{n}\psi_{i}\log\left(\frac{1}{\psi_{i}}\right),$$

where $\psi_{1},\psi_{2},\ldots ,\psi_{n}\in {ℜ}^{+}$ with $\sum_{i=1}^{n}\psi_{i}=1$.

There are many applications of the Shannon entropy in most applied sciences and in other sciences, such as biology [3], genomic geography [4], and finance [5]. Currently, the Shannon entropy is applied in the simulation of laser dynamics and as an objective measure to evaluate models and compare observational results [6,7].

In 1932, George Zipf gave the idea that the size of the $r^{\prime }$th largest occurrence of an event is inversely proportional to its rank. That is, this law states that $P_{r}=1/r^{b}$, where $P_{r}$ is the frequency of occurrence of the $r^{\prime }$th ranked and **b** is close to unity. As in linguistics, Zipf found that one can calculate the number of times each word appears in the text. Therefore, if the rank (γ) of the word is in accordance with the frequency of the word’s appearance (ρ), then the product of these two numbers is a constant ((C): C=ρ.γ, see [8,9]).

There are several applications of the Zipf law, and here we present some of them. This law has been used in city populations. Kristian Giesen and Jens Suedekum conducted a study on the city measure distributions of single German districts’ reliance on researching the phenomenon [10]. They built their study based on the intuition by Gabaix (1999) which states that the Zipf law takes after an irregular development process. This means that Gabaix displays that if the districts follow the Gibrat law, they should notice the Zipf law at both the districts at a national level. By utilizing non-parametric procudures, they found that the Gibrat law holds in each German district, regardless of how “districts” are defined. To put it differently, the Gibrat and Zipf laws are inclined to hold ubiquitously in space. In geology, the Zipf law has been used with temperate prosperity in the resource estimation of extracting ores from the ground and petroleum [11]. In principle, it forecasts how many entities of a confident size can be left in a sequence of decreasing size, assuming the largest has been established. The solar flare intensity (M. E. J. Newman, 2004) [12] represents the cumulative distribution of the vertex gamma-ray density of solar flares, for which perceptions were made between 1980 and 1989 by the well-known HardX-Ray fulmination spectrometer onboard the solar maximum mission satellite launched in 1980. The spectrometer uses a CsI gleaming discloser to measure gamma-rays from solar flares. For website traffic (Shane Parkins, 2015) [13], the Zipf law seems, by all accounts, to be the control as opposed to the special case. It is available at the level of routers that transmit data from one geographic location to another and in the content of the World Wide Web. At the social and economic levels, it also determines how persons choose the sites they visit and form peer-to-peer societies. The omnipresent nature of the Zipf law in cyberspace is geared toward deeper empathy with the internet phenomena, for example, discovering the potential of prevalence proxy caches in divergent Autonomous Systems (ASes) with the purpose of reducing the costs incurred by internet service providers and pacification of the load on the internet backbone [14].

It was determined that Zipf’s law can describe the size and rank distribution of earthquakes, including those with magnitude, but it cannot predict when they will occur. In the earth-moon the crater size-frequency distribution can be represented by the Zipf law [12,15].

In 1966, Benoit Mandelbrot gave an enhancement for the Zipf law, known as **ZML**, which gives a generalization of the account of the low-rank words in corpus [16]:

$$g\left(i\right)=\frac{c}{{\left(i+h\right)}^{r}},$$

where i<1000,r,c>0 and if h=0, we get the Zipf law.

If n∈N, h≥0, r>0 and i∈{1,2,…,n}, then the Zipf–Mandelbrot law (probability mass function) is defined by

$$G\left(i,n,h,r\right)=\frac{1/{\left(i+h\right)}^{r}}{Q_{n,h,r}}.$$

The formula for **ZME** is given by

(2) $$Z\left(Q,h,r\right)=\frac{r}{Q_{n,h,r}}\sum_{i=1}^{n}\frac{\log(i+h)}{{\left(i+h\right)}^{r}}+\log Q_{n,h,r},$$

where $Q_{n,h,r}=\sum_{i=1}^{n}\frac{1}{{\left(i+h\right)}^{r}}$.

There are many applications of **ZML** which can be found in linguistics [16,17], ecological field studies [18], and information sciences [9]. Recently, the Zipf–Mandelbrot law was applied to various types of *f*-divergences and distances, for example Kullback–Leibler divergence, Bhattacharyya distance (via coefficient), Hellinger distance, $x^{2}$-divergence, etc [19].

To complete this section, we give some notions and results from ref. [20].

Let g:G⟶R be a convex function defined on the convex set G, $T_{n}=\left\{1,2,3,\ldots ,n\right\}$. Let *s* be fixed positive integer and *l* be all those positive integers, such that 1≤l≤s≤n. Suppose $M_{1}^{s},M_{2}^{s},\ldots ,M_{l}^{s}$ represents any subsets of $T_{n}$, such that $M_{1}^{s}⋃M_{2}^{s}\ldots ⋃M_{l}^{s}=T_{n}$ and $M_{i}^{s}⋂M_{j}^{s}=\emptyset$ for i≠j, where $i,j\in T_{n}$, $x_{i}\in G$, $\psi_{i}>0$ with $\sum_{i=1}^{n}\psi_{i}=1$, and for any M⊆{1,2,…,n}, M≠∅, we represent $\Psi_{M}=:\sum_{i\in M}\psi_{i}$ and $\Omega_{M}=:\sum_{i\in M}\omega_{i}$ for positive real numbers $\psi_{i},\omega_{i}$. For the convex function *g* and the *n*-tuples $x=(x_{1},x_{2},\ldots ,x_{n})$, $\psi =(\psi_{1},\psi_{2},\ldots ,\psi_{n})$, the following functional is defined:

(3) $$A_{s}=\max_{M_{1}^{s},M_{2}^{s},\ldots ,M_{l}^{s}}\left[\Psi_{M_{1}^{s}}g\left(\frac{1}{\Psi_{M_{1}^{s}}}\sum_{i\in M_{1}^{s}}\psi_{i}x_{i}\right)+\Psi_{M_{2}^{s}}g\left(\frac{1}{\Psi_{M_{2}^{s}}}\sum_{i\in M_{2}^{s}}\psi_{i}x_{i}\right)+\ldots +\Psi_{M_{l}^{s}}g\left(\frac{1}{\Psi_{M_{l}^{s}}}\sum_{i\in M_{l}^{s}}\psi_{i}x_{i}\right)\right].$$

Particularly, for s=2,3, we have

$$\begin{array}{c} A_{2}=\;\max_{M_{1}^{2},M_{2}^{2}}\left[\Psi_{M_{1}^{2}}g\left(\frac{1}{\Psi_{M_{1}^{2}}}\sum_{i\in M_{1}^{2}}\psi_{i}x_{i}\right)+\Psi_{M_{2}^{2}}g\left(\frac{1}{\Psi_{M_{2}^{2}}}\sum_{i\in M_{2}^{2}}\psi_{i}x_{i}\right)\right]\\ A_{3}=\max_{M_{1}^{3},M_{2}^{3},M_{3}^{3}}\left[\Psi_{M_{1}^{3}}g\left(\frac{1}{\Psi_{M_{1}^{3}}}\sum_{i\in M_{1}^{3}}\psi_{i}x_{i}\right)+\Psi_{M_{2}^{3}}g\left(\frac{1}{\Psi_{M_{2}^{3}}}\sum_{i\in M_{2}^{3}}\psi_{i}x_{i}\right)+\Psi_{M_{3}^{3}}g\left(\frac{1}{\Psi_{M_{3}^{3}}}\sum_{i\in M_{3}^{3}}\psi_{i}x_{i}\right)\right]. \end{array}$$

Analogously, for other particular values of *s* with 1≤l≤s≤n, one can obtain different functionals.

The following generalized refinements of the Jensen inequality were given in refs. [20,21].

**Theorem** **1** ([20]). *Let g:M⟶R be a convex function defined on the convex set M. If $x_{i}\in M$ and $\psi_{i}>0$ for $i,k\in T_{n}$ with $\sum_{i=1}^{n}\psi_{i}=1$, then we have*

(4) $$\sum_{i=1}^{n}\psi_{i}g\left(x_{i}\right)\geq A_{k}\geq A_{k-1}\geq \ldots \geq A_{3}\geq A_{2}\geq g\left(\sum_{i=1}^{n}\psi_{i}x_{i}\right).$$

**Theorem** **2** ([21]). *Let g:M⟶R be a convex function defined on the convex set M, $x_{j}\in M$, and $\psi_{j}>0$ for j∈{1,…,n} with $\sum_{j=1}^{n}\psi_{j}=1$. Then,*

(5) $$\begin{array}{cc} g\left(\sum_{j=1}^{n}\psi_{j}x_{j}\right) & \leq \;\min_{k\in T_{n}}\left[\left(1-\psi_{k}\right)g\left(\frac{\sum_{j=1}^{n}\psi_{j}x_{j}-\psi_{k}x_{k}}{1-\psi_{k}}\right)+\psi_{k}g\left(x_{k}\right)\right]\\ & \leq \;\frac{1}{n}\left[\sum_{k=1}^{n}\left(1-\psi_{k}\right)g\left(\frac{\sum_{j=1}^{n}\psi_{j}x_{j}-\psi_{k}x_{k}}{1-\psi_{k}}\right)+\sum_{k=1}^{n}\psi_{k}g\left(x_{k}\right)\right]\\ & \leq \;\max_{k\in T_{n}}\left[\left(1-\psi_{k}\right)g\left(\frac{\sum_{j=1}^{n}\psi_{j}x_{j}-\psi_{k}x_{k}}{1-\psi_{k}}\right)+\psi_{k}g\left(x_{k}\right)\right]\\ & \leq \;\sum_{j=1}^{n}\psi_{j}g\left(x_{j}\right). \end{array}$$

For some other results related to the Jensen inequality and the Shannon and Zipf–Mandelbrot entropies, see refs. [8,22,23,24].

Due to the great importance of the Shannon and Zipf–Mandelbrot entropies, many results are devoted to these entropies in the literature. The main focus of this paper was to associate some refinements of the Jensen inequality to the Shannon and Zipf–Mandelbrot entropies. In this paper, we use the main results given in ref. [20] and obtain some estimations for these entropies. We also discuss some particular cases of these results. At the end of the paper, we give some applications in linguistics. The idea of this paper can be applied for other results of the Jensen inequality to obtain new estimations for these entropies.

## 2. Estimations for the Shannon Entropy

We start by giving our first main result for the Shannon entropy.

**Theorem** **3.** *Let $\psi_{i},\omega_{i}\in R^{+}$, where i=1,2,…,n, with $\sum_{i=1}^{n}\psi_{i}=1$. Then, the following inequalities hold:*

(6) $$\begin{array}{cc} -S\left(\Psi \right)-\sum_{i=1}^{n}\psi_{i}\log\omega_{i} & \geq \;\max_{M_{1}^{k},M_{2}^{k},\ldots ,M_{l}^{k}}\left\{\Psi_{M_{1}^{k}}\log\left(\frac{\Psi_{M_{1}^{k}}}{\Omega_{M_{1}^{k}}}\right)+\ldots +\Psi_{M_{l}^{k}}\log\left(\frac{\Psi_{M_{l}^{k}}}{\Omega_{M_{l}^{k}}}\right)\right\}\\ & \geq \;\max_{M_{1}^{k},M_{2}^{k},\ldots ,M_{l-1}^{k}}\left\{\Psi_{M_{1}^{k}}\log\left(\frac{\Psi_{M_{1}^{k}}}{\Omega_{M_{1}^{k}}}\right)+\ldots +\Psi_{M_{l-1}^{k}}\log\left(\frac{\Psi_{M_{l-1}^{k}}}{\Omega_{M_{l-1}^{k}}}\right)\right\}\\ & \geq \;\ldots \geq \log\left(\frac{1}{\Omega_{T_{n}}}\right). \end{array}$$

**Proof.**  If we take g(x)=−logx, $x_{i}=\frac{\omega_{i}}{\psi_{i}}$, $i\in T_{n}$ in (4), then we obtain

$$\begin{array}{ccc} \sum_{i=1}^{n}\psi_{i}g\left(x_{i}\right) & = & -\sum_{i=1}^{n}\psi_{i}\log\left(\frac{\omega_{i}}{\psi_{i}}\right)=\sum_{i=1}^{n}\psi_{i}\log\psi_{i}-\sum_{i=1}^{n}\psi_{i}\log\omega_{i}\\ & = & -S\left(\Psi \right)-\sum_{i=1}^{n}\psi_{i}\log\omega_{i} \end{array}$$

and

$$\begin{array}{ccc} A_{k} & = & \max_{M_{1}^{k},M_{2}^{k},\ldots ,M_{l}^{k}}\left\{\Psi_{M_{1}^{k}}\log\left(\frac{\Psi_{M_{1}^{k}}}{\Omega_{M_{1}^{k}}}\right)+\ldots +\Psi_{M_{l}^{k}}\log\left(\frac{\Psi_{M_{l}^{k}}}{\Omega_{M_{l}^{k}}}\right)\right\}.\\ A_{k-1} & = & \max_{M_{1}^{k},M_{2}^{k},\ldots ,M_{l-1}^{k}}\left\{\Psi_{M_{1}^{k}}\log\left(\frac{\Psi_{M_{1}^{k}}}{\Omega_{M_{1}^{k}}}\right)+\ldots +\Psi_{M_{l-1}^{k}}\log\left(\frac{\Psi_{M_{l-1}^{k}}}{\Omega_{M_{l-1}^{k}}}\right)\right\}\\ \vdots \\ A_{3} & = & \max_{M_{1}^{3},M_{2}^{3},M_{3}^{3}}\left\{\Psi_{M_{1}^{3}}\log\left(\frac{\Psi_{M_{1}^{3}}}{\Omega_{M_{1}^{3}}}\right)+\Psi_{M_{2}^{3}}\log\left(\frac{\Psi_{M_{2}^{3}}}{\Omega_{M_{2}^{3}}}\right)+\Psi_{M_{3}^{3}}\log\left(\frac{\Psi_{M_{3}^{3}}}{\Omega_{M_{3}^{3}}}\right)\right\}.\\ A_{2} & = & \max_{M_{1}^{2},M_{2}^{2}}\left\{\Psi_{M_{1}^{2}}\log\left(\frac{\Psi_{M_{1}^{2}}}{\Omega_{M_{1}^{2}}}\right)+\Psi_{M_{2}^{2}}\log\left(\frac{\Psi_{M_{2}^{2}}}{\Omega_{M_{2}^{2}}}\right)\right\}. \end{array}$$

Therefore, from (4), we deduce (6). ☐

**Corollary** **1.** *Let $\psi_{i},\omega_{i}\in R^{+}$, where i=1,2,…,n, with $\sum_{i=1}^{n}\psi_{i}=1$, then*

(7) $$\begin{array}{ccc} -S\left(\Psi \right)-\sum_{i=1}^{n}\psi_{i}\log\omega_{i} & \geq & \max_{M_{1}^{2},M_{2}^{2}}\left\{\Psi_{M_{1}^{2}}\log\left(\frac{\Psi_{M_{1}^{2}}}{\Omega_{M_{1}^{2}}}\right)+\Psi_{M_{2}^{2}}\log\left(\frac{\Psi_{M_{2}^{2}}}{\Omega_{M_{2}^{2}}}\right)\right\}\geq \log\left(\frac{1}{\Omega_{T_{n}}}\right). \end{array}$$

**Proof.**  By taking k=2 in (6), we obtain (7). ☐

**Corollary** **2.** *Let $\psi_{i},\omega_{i}\in R^{+}$, $i\in T_{n}$ with $\sum_{i=1}^{n}\psi_{i}=1$, then*

(8) $$\begin{array}{cc} -S\left(\Psi \right)-\sum_{i=1}^{n}\psi_{i}\log\omega_{i} & \geq \;\max_{k\in T_{n}}\left\{\psi_{k}\log\left(\frac{\psi_{k}}{\omega_{k}}\right)+\left(1-\psi_{k}\right)\log\left(\frac{1-\psi_{k}}{\sum_{i=1}^{n}\omega_{i}-\omega_{k}}\right)\right\}\\ & \geq \;\log\left(\frac{1}{\Omega_{T_{n}}}\right). \end{array}$$

**Proof.**  If we take $M_{1}^{2}=\left\{k\right\}$ in (7), then obviously, $M_{2}^{2}=\left\{1,2,\ldots ,n\right\}\backslash \left\{k\right\}$ and $\Psi_{M_{1}^{2}}=\psi_{k},\Psi_{M_{2}^{2}}=1-\psi_{k}$. Hence, from (7), we deduce (8). ☐

**Remark** **1.** *Note that Corollary 1 is, in fact, the application of ([25], Theorem 1) and Corollary 2 is the application of ([21], Theorem 1).*

In the following corollary, we discuss another particular case of Theorem 3.

**Corollary** **3.** *Let $\psi_{i},\omega_{i}\in R^{+}$ for $i\in T_{n}$ with $\sum_{i=1}^{n}\psi_{i}=1$, then*

(9) $$\begin{array}{c} -S\left(\Psi \right)\geq \Psi_{M_{1}^{2}}\log\left(\frac{\Psi_{M_{1}^{2}}}{n}\right)+\Psi_{M_{2}^{2}}\log\left(\frac{\Psi_{M_{2}^{2}}}{n}\right)\geq 0. \end{array}$$

**Proof.**  By taking $\omega_{i}=1$ in (7), i=1,2,…,n, we get (9). ☐

**Remark** **2.** *It is obvious that*

$$\begin{array}{c} \max_{M_{1}^{2},M_{2}^{2}}\left\{\Psi_{M_{1}^{2}}\log\left(\frac{\Psi_{M_{1}^{2}}}{\Omega_{M_{1}^{2}}}\right)+\Psi_{M_{2}^{2}}\log\left(\frac{\Psi_{M_{2}^{2}}}{\Omega_{M_{2}^{2}}}\right)\right\}\\ \;\geq \max_{k\in T_{n}}\left\{\psi_{k}\log\left(\frac{\psi_{k}}{\omega_{k}}\right)+\left(1-\psi_{k}\right)\log\left(\frac{1-\psi_{k}}{\sum_{i=1}^{n}\omega_{i}-\omega_{k}}\right)\right\}. \end{array}$$

## 3. Estimations for the Zipf–Mandelbrot Entropy

In the following main result, we obtain some general estimations for the Zipf–Mandelbrot entropy.

**Theorem** **4.** *Let n∈N, h≥0, r>0, $\omega_{i}>0$, $i\in T_{n}$, then*

(10) $$\begin{array}{cc} -Z(Q,h,r) & -\;\sum_{i=1}^{n}\frac{\log\omega_{i}}{{\left(i+h\right)}^{r}Q_{n,h,r}}\\ & \geq \;\max_{M_{1}^{k},M_{2}^{k},\ldots ,M_{l}^{k}}\{\sum_{i\in M_{1}^{k}}\frac{1}{{\left(i+h\right)}^{r}Q_{n,h,r}}\log\left(\frac{\sum_{i\in M_{1}^{k}}\frac{1}{{\left(i+h\right)}^{r}Q_{n,h,r}}}{\sum_{i\in M_{1}^{k}}\omega_{i}}\right)+\\ & \;\;\ldots +\sum_{i\in M_{l}^{k}}\frac{1}{{\left(i+h\right)}^{r}Q_{n,h,r}}\log\left(\frac{\sum_{i\in M_{l}^{k}}\frac{1}{{\left(i+h\right)}^{r}Q_{n,h,r}}}{\sum_{i\in M_{l}^{k}}\omega_{i}}\right)\}\\ & \geq \;\max_{M_{1}^{k},M_{2}^{k},\ldots ,M_{l-1}^{k}}\{\sum_{i\in M_{1}^{k}}\frac{1}{{\left(i+h\right)}^{r}Q_{n,h,r}}\log\left(\frac{\sum_{i\in M_{1}^{k}}\frac{1}{{\left(i+h\right)}^{r}Q_{n,h,r}}}{\sum_{i\in M_{1}^{k}}\omega_{i}}\right)+\\ & \;\;\ldots +\sum_{i\in M_{l-1}^{k}}\frac{1}{{\left(i+h\right)}^{r}Q_{n,h,r}}\log\left(\frac{\sum_{i\in M_{l-1}^{k}}\frac{1}{{\left(i+h\right)}^{r}Q_{n,h,r}}}{\sum_{i\in M_{l-1}^{k}}\omega_{i}}\right)\}\\ & \geq \;\ldots \geq \log\left(\frac{1}{\Omega_{T_{n}}}\right). \end{array}$$

**Proof.**  If we substitute $\psi_{i}$ with $\frac{1}{{\left(i+h\right)}^{r}Q_{n,h,r}}$, $\left(i\in T_{n}\right)$, we have

$$\begin{array}{ccc} \sum_{i=1}^{n}\psi_{i}g\left(x_{i}\right) & = & -\sum_{i=1}^{n}\psi_{i}\log\omega_{i}+\sum_{i=1}^{n}\psi_{i}\log\psi_{i}\\ & = & \sum_{i=1}^{n}\frac{1}{{\left(i+h\right)}^{r}Q_{n,h,r}}\log\frac{1}{{\left(i+h\right)}^{r}Q_{n,h,r}}-\sum_{i=1}^{n}\frac{\log\omega_{i}}{{\left(i+h\right)}^{r}Q_{n,h,r}}\\ & = & -\sum_{i=1}^{n}\frac{\log[{\left(i+h\right)}^{r}Q_{n,h,r}]}{{\left(i+h\right)}^{r}Q_{n,h,r}}-\sum_{i=1}^{n}\frac{\log\omega_{i}}{{\left(i+h\right)}^{r}Q_{n,h,r}}\\ & = & -\sum_{i=1}^{n}\frac{r\log(i+h)}{{\left(i+h\right)}^{r}Q_{n,h,r}}-\sum_{i=1}^{n}\frac{\log Q_{n,h,r}}{{\left(i+h\right)}^{r}Q_{n,h,r}}-\sum_{i=1}^{n}\frac{\log\omega_{i}}{{\left(i+h\right)}^{r}Q_{n,h,r}}\\ & = & -\frac{r}{Q_{n,h,r}}\sum_{i=1}^{n}\frac{\log(i+h)}{{\left(i+h\right)}^{r}}-\frac{\log Q_{n,h,r}}{Q_{n,h,r}}\sum_{i=1}^{n}\frac{1}{{\left(i+h\right)}^{r}}-\sum_{i=1}^{n}\frac{\log\omega_{i}}{{\left(i+h\right)}^{r}Q_{n,h,r}}. \end{array}$$

Then,

$$\sum_{i=1}^{n}\psi_{i}g\left(x_{i}\right)=-Z\left(Q,h,r\right)-\sum_{i=1}^{n}\frac{\log\omega_{i}}{{\left(i+h\right)}^{r}Q_{n,h,r}},$$

where $Q_{n,h,r}=\sum_{i=1}^{n}\frac{1}{{\left(i+h\right)}^{r}}$ and $\sum_{i=1}^{n}\frac{1}{{\left(i+h\right)}^{r}Q_{n,h,r}}=1$.

Now, by applying Theorem 3 for $\psi_{i}=\frac{1}{{\left(i+h\right)}^{r}Q_{n,h,r}}$, we obtain the required result. ☐

**Corollary** **4.** *Let n∈N, h≥0, r>0, $\omega_{i}>0$ for $i\in T_{n}$, then*

(11) $$\begin{array}{cc} -Z(Q,h,r) & -\;\sum_{i=1}^{n}\frac{\log\omega_{i}}{{\left(i+h\right)}^{r}Q_{n,h,r}}\\ \geq \max_{M_{1}^{2},M_{2}^{2}} & \{\;\sum_{i\in M_{1}^{2}}\frac{1}{{\left(i+h\right)}^{r}Q_{n,h,r}}\log\left(\frac{\sum_{i\in M_{1}^{2}}\frac{1}{{\left(i+h\right)}^{r}Q_{n,h,r}}}{\sum_{i\in M_{1}^{2}}\omega_{i}}\right)\\ & +\;\sum_{i\in M_{2}^{2}}\frac{1}{{\left(i+h\right)}^{r}Q_{n,h,r}}\log\left(\frac{\sum_{i\in M_{2}^{2}}\frac{1}{{\left(i+h\right)}^{r}Q_{n,h,r}}}{\sum_{i\in M_{2}^{2}}\omega_{i}}\right)\}\geq \log\left(\frac{1}{\Omega_{T_{n}}}\right). \end{array}$$

**Proof.**  By taking k=2 in (10), we obtain (11). ☐

We can use Theorem 4 to obtain the following corollary.

**Corollary** **5.** *Let n∈N, h≥0, r>0, then*

(12) $$\begin{array}{cc} -Z\left(Q,h,r\right)\geq \max_{M_{1}^{2},M_{2}^{2}} & \{\;\sum_{i\in M_{1}^{2}}\frac{1}{{\left(i+h\right)}^{r}Q_{n,h,r}}\log\left(\frac{\sum_{i\in M_{1}^{2}}\frac{1}{{\left(i+h\right)}^{r}Q_{n,h,r}}}{\sum_{i\in M_{1}^{2}}1}\right)\\ & +\;\sum_{i\in M_{2}^{2}}\frac{1}{{\left(i+h\right)}^{r}Q_{n,h,r}}\log\left(\frac{\sum_{i\in M_{2}^{2}}\frac{1}{{\left(i+h\right)}^{r}Q_{n,h,r}}}{\sum_{i\in M_{2}^{2}}1}\right)\}\geq \log\left(\frac{1}{n}\right). \end{array}$$

**Proof.**  By taking $\omega_{i}=1$ in (11), i=1,2,…,n, we get (12). ☐

**Corollary** **6.** *Let n∈N, h≥0, r>0, then*

$$\begin{array}{c} -Z\left(Q,h,r\right)-\sum_{i=1}^{n}\frac{\log\omega_{i}}{{\left(i+h\right)}^{r}Q_{n,h,r}}\geq \max_{k\in T_{n}}\{\frac{1}{{\left(k+h\right)}^{r}Q_{n,h,r}}\log\left(\frac{1}{{\left(k+h\right)}^{r}Q_{n,h,r}\omega_{k}}\right)\\ \;+\left(\frac{{\left(k+h\right)}^{r}Q_{n,h,r}-1}{{\left(k+h\right)}^{r}Q_{n,h,r}}\right)\log\left(\frac{\left({\left(k+h\right)}^{r}Q_{n,h,r}-1\right)/{\left(k+h\right)}^{r}Q_{n,h,r}}{\Omega_{T_{n}}-\omega_{k}}\right)\}\\ \;\geq \log\left(\frac{1}{n}\right).\; \end{array}$$

**Proof.**  Using $M_{1}^{2}=\left\{k\right\}$ in (12), we obtain Corollary 6. ☐

**Remark** **3.** *Note that Corollary 4 is in fact the application of ([25], Theorem 1), and Corollary 6 is the application of ([21], Theorem 1).*

**Remark** **4.** *By using Remark 2, we also have*

$$\begin{array}{c} \max_{M_{1}^{2},M_{2}^{2}}\left\{\sum_{i\in M_{1}^{2}}\frac{1}{{\left(i+h\right)}^{r}Q_{n,h,r}}\log\left(\frac{\sum_{i\in M_{1}^{2}}\frac{1}{{\left(i+h\right)}^{r}Q_{n,h,r}}}{\sum_{i\in M_{1}^{2}}\omega_{i}}\right)+\sum_{i\in M_{2}^{2}}\frac{1}{{\left(i+h\right)}^{r}Q_{n,h,r}}\log\left(\frac{\sum_{i\in M_{2}^{2}}\frac{1}{{\left(i+h\right)}^{r}Q_{n,h,r}}}{\sum_{i\in M_{2}^{2}}\omega_{i}}\right)\right\}\\ \;\geq \max_{k\in T_{n}}\{\frac{1}{{\left(k+h\right)}^{r}Q_{n,h,r}}\log\left(\frac{1}{{\left(k+h\right)}^{r}Q_{n,h,r}\omega_{k}}\right)\\ \;+\left(\frac{{\left(k+h\right)}^{r}Q_{n,h,r}-1}{{\left(k+h\right)}^{r}Q_{n,h,r}}\right)\log\left(\frac{\left({\left(k+h\right)}^{r}Q_{n,h,r}-1\right)/{\left(k+h\right)}^{r}Q_{n,h,r}}{\Omega_{T_{n}}-\omega_{k}}\right)\}. \end{array}$$

In the following result, we obtain the estimation for the Zipf–Mandelbrot entropy concerning two different parameters.

**Theorem** **5.** *Let u,v≥0, $r_{1},r_{2}>0$, then*

(13) $$\begin{array}{cc} -Z(Q,u,r_{1}) & +\;\sum_{i=1}^{n}\frac{\log({\left(i+v\right)}^{r_{2}}Q_{n,v,r_{2}})}{{\left(i+u\right)}^{r_{1}}Q_{n,u,r_{1}}}\\ & \geq \;\max_{M_{1}^{k},M_{2}^{k},\ldots ,M_{l}^{k}}\{\sum_{i\in M_{1}^{k}}\frac{1}{{\left(i+u\right)}^{r_{1}}Q_{n,u,r_{1}}}\log\left(\frac{\sum_{i\in M_{1}^{k}}\frac{1}{{\left(i+u\right)}^{r_{1}}Q_{n,u,r_{1}}}}{\sum_{i\in M_{1}^{k}}\frac{1}{{\left(i+v\right)}^{r_{2}}Q_{n,v,r_{2}}}}\right)+\\ & \;\;\ldots +\sum_{i\in M_{l}^{k}}\frac{1}{{\left(i+u\right)}^{r_{1}}Q_{n,u,r_{1}}}\log\left(\frac{\sum_{i\in M_{l}^{k}}\frac{1}{{\left(i+u\right)}^{r_{1}}Q_{n,u,r_{1}}}}{\sum_{i\in M_{l}^{k}}\frac{1}{{\left(i+v\right)}^{r_{2}}Q_{n,v,r_{2}}}}\right)\}\\ & \geq \;\max_{M_{1}^{k},M_{2}^{k},\ldots ,M_{l-1}^{k}}\{\sum_{i\in M_{1}^{k}}\frac{1}{{\left(i+u\right)}^{r_{1}}Q_{n,u,r_{1}}}\log\left(\frac{\sum_{i\in M_{1}^{k}}\frac{1}{{\left(i+u\right)}^{r_{1}}Q_{n,u,r_{1}}}}{\sum_{i\in M_{1}^{k}}\frac{1}{{\left(i+v\right)}^{r_{2}}Q_{n,v,r_{2}}}}\right)+\\ & \;\;\ldots +\sum_{i\in M_{l-1}^{k}}\frac{1}{{\left(i+u\right)}^{r_{1}}Q_{n,u,r_{1}}}\log\left(\frac{\sum_{i\in M_{l-1}^{k}}\frac{1}{{\left(i+u\right)}^{r_{1}}Q_{n,u,r_{1}}}}{\sum_{i\in M_{l-1}^{k}}\frac{1}{{\left(i+v\right)}^{r_{2}}Q_{n,v,r_{2}}}}\right)\}\\ & \geq \;\ldots \geq 0. \end{array}$$

**Proof.**  Let $\psi_{i}=\frac{1}{{\left(i+u\right)}^{r_{1}}Q_{n,u,r_{1}}}$, $\omega_{i}=\frac{1}{{\left(i+v\right)}^{r_{2}}Q_{n,v,r_{2}}}$, $i\in T_{n}$. Then, using the proof of Theorem 4, we get

$$\begin{array}{ccc} \sum_{i=1}^{n}\psi_{i}\log\psi_{i} & = & \sum_{i=1}^{n}\frac{1}{{\left(i+u\right)}^{r_{1}}Q_{n,u,r_{1}}}\log\frac{1}{{\left(i+u\right)}^{r_{1}}Q_{n,u,r_{1}}}=-Z\left(Q,u,r_{1}\right),\\ -\sum_{i=1}^{n}\psi_{i}\log\omega_{i} & = & \sum_{i=1}^{n}\frac{1}{{\left(i+u\right)}^{r_{1}}Q_{n,u,r_{1}}}\log\frac{1}{{\left(i+v\right)}^{r_{2}}Q_{n,v,r_{2}}}\\ & = & \sum_{i=1}^{n}\frac{\log({\left(i+v\right)}^{r_{2}}Q_{n,v,r_{2}})}{{\left(i+u\right)}^{r_{1}}Q_{n,u,r_{1}}},\\ \sum_{i=1}^{n}\omega_{i} & = & \sum_{i=1}^{n}\frac{1}{{\left(i+v\right)}^{r_{2}}Q_{n,v,r_{2}}}=1. \end{array}$$

Therefore, using (6) for $\psi_{i}=\frac{1}{{\left(i+u\right)}^{r_{1}}Q_{n,u,r_{1}}}$ and $\omega_{i}=\frac{1}{{\left(i+v\right)}^{r_{2}}Q_{n,v,r_{2}}}$, $i\in T_{n}$, we obtain (13). ☐

**Corollary** **7.** *Let n∈N, u,v≥0, $r_{1},r_{2}>0$, then*

(14) $$\begin{array}{c} -Z(Q,u,r_{1})+\;\sum_{i=1}^{n}\frac{\log({\left(i+v\right)}^{r_{2}}Q_{n,v,r_{2}})}{{\left(i+u\right)}^{r_{1}}Q_{n,u,r_{1}}}\\ \;\geq \max_{M_{1}^{2},M_{2}^{2}}\{\;\sum_{i\in M_{1}^{2}}\frac{1}{{\left(i+u\right)}^{r_{1}}Q_{n,u,r_{1}}}\log\left(\frac{\sum_{i\in M_{1}^{2}}\frac{1}{{\left(i+u\right)}^{r_{1}}Q_{n,u,r_{1}}}}{\sum_{i\in M_{1}^{2}}\frac{1}{{\left(i+v\right)}^{r_{2}}Q_{n,v,r_{2}}}}\right)\\ \;+\;\sum_{i\in M_{2}^{2}}\frac{1}{{\left(i+u\right)}^{r}Q_{n,u,r_{1}}}\log\left(\frac{\sum_{i\in M_{2}^{2}}\frac{1}{{\left(i+u\right)}^{r_{1}}Q_{n,u,r_{1}}}}{\sum_{i\in M_{2}^{2}}\frac{1}{{\left(i+v\right)}^{r_{2}}Q_{n,v,r_{2}}}}\right)\}\geq 0. \end{array}$$

**Proof.**  By taking k=2 in (13), we obtain (14). ☐

**Corollary** **8.** *Let u,v≥0, $r_{1},r_{2}>0$, then*

(15) $$\begin{array}{cc} -Z(Q,u,r_{1}) & +\;\sum_{i=1}^{n}\frac{\log({\left(i+v\right)}^{r_{2}}Q_{n,v,r_{2}})}{{\left(i+u\right)}^{r_{1}}Q_{n,u,r_{1}}}\\ & \geq \;\max_{k\in T_{n}}\{\frac{1}{{\left(k+u\right)}^{r_{1}}Q_{n,u,r_{1}}}\log\left(\frac{{\left(k+v\right)}^{r_{2}}Q_{n,v,r_{2}}}{{\left(k+u\right)}^{r_{1}}Q_{n,u,r_{1}}}\right)\\ & +\;\left(\frac{{\left(k+u\right)}^{r_{1}}Q_{n,u,r_{1}}-1}{{\left(k+u\right)}^{r_{1}}Q_{n,u,r_{1}}}\right)\\ & \log\;\left(\frac{\left({\left(k+u\right)}^{r_{1}}Q_{n,u,r_{1}}-1\right)/{\left(k+u\right)}^{r_{1}}Q_{n,u,r_{1}}}{\left({\left(k+v\right)}^{r_{2}}Q_{n,v,r_{2}}-1\right)/{\left(k+v\right)}^{r_{2}}Q_{n,v,r_{2}}}\right)\}\\ & \geq \;0. \end{array}$$

**Proof.**  Using $M_{1}^{2}=\left\{k\right\}$ in (14), we obtain (15). ☐

Now we give applications of the above results in linguistics.

In ref. [26], Gelbukh and Sidorov observed the difference between the coefficients $r_{1}$ and $r_{2}$ in the Zipf law for the English and Russian languages. They processed 39 literature texts for each language, chosen randomly from different genres, with the requirement that the size be greater than 10,000 running words each. They calculated the coefficients for each of the mentioned texts and as a result, they obtained an average of $r_{1}=0.973863$ for the English language and $r_{2}=0.892869$ for the Russian language.

In the following results, we give the application of inequality (11) for the English language.

**Application** **1.** *Let n∈N, $\omega_{i}>0$ for $i\in T_{n}$ Then, we have*

(16) $$\begin{array}{c} -Z(Q,0,0.973863)-\;\sum_{i=1}^{n}\frac{\log\omega_{i}}{i^{0.973863}Q_{n,0,0.973863}}\\ \;\geq \max_{M_{1}^{2},M_{2}^{2}}\{\;\sum_{i\in M_{1}^{2}}\frac{1}{i^{0.973863}Q_{n,0,0.973863}}\log\left(\frac{\sum_{i\in M_{1}^{2}}\frac{1}{i^{0.973863}Q_{n,0,0.973863}}}{\sum_{i\in M_{1}^{2}}\omega_{i}}\right)\\ \;+\;\sum_{i\in M_{2}^{2}}\frac{1}{i^{0.973863}Q_{n,0,0.973863}}\log\left(\frac{\sum_{i\in M_{2}^{2}}\frac{1}{i^{0.973863}Q_{n,0,0.973863}}}{\sum_{i\in M_{2}^{2}}\omega_{i}}\right)\}\geq \log\left(\frac{1}{\Omega_{T_{n}}}\right). \end{array}$$

Similarly, we can give an application for the Russian language. 

Now we give an application for the result of the related two parameters: $r_{1}=0.973863$ for the English language and $r_{2}=0.892869$ for the Russian language, which is in fact application of the inequality (14).

**Application** **2.** *Let n∈N. Then, we have*

$$\begin{array}{c} -Z(Q,0,0.973863)+\sum_{i=1}^{n}\frac{\log(i^{0.892869}Q_{n,0,0.892869})}{i^{0.973863}Q_{n,0,0.973863}}\\ \;\geq \max_{M_{1}^{2},M_{2}^{2}}\{\sum_{i\in M_{1}^{2}}\frac{1}{i^{0.973863}Q_{n,0,0.973863}}\log\left(\frac{\sum_{i\in M_{1}^{2}}\frac{1}{i^{0.892869}Q_{n,0,0.892869}}}{\sum_{i\in M_{1}^{2}}\frac{1}{i^{0.973863}Q_{n,0,0.973863}}}\right)\\ \;+\sum_{i\in M_{2}^{2}}\frac{1}{i^{0.973863}Q_{n,0,0.973863}}\log\left(\frac{\sum_{i\in M_{2}^{2}}\frac{1}{i^{0.892869}Q_{n,0,0.892869}}}{\sum_{i\in M_{2}^{2}}\frac{1}{i^{0.973863}Q_{n,0,0.973863}}}\right)\}\geq 0. \end{array}$$

**Remark** **5.** *In a similar way, applications of the remaining results from Section 3 can be given.*
